# Transcription regulators and ultra-rare and other rare translocation-related sarcomas treated with trabectedin: A proof of principle from a *post-hoc* analysis

**DOI:** 10.3389/fonc.2022.1042479

**Published:** 2022-12-08

**Authors:** Emanuela Palmerini, Roberta Sanfilippo, Giovanni Grignani, Angela Buonadonna, Antonella Romanini, Giuseppe Badalamenti, Virginia Ferraresi, Bruno Vincenzi, Alessandro Comandone, Antonio Pizzolorusso, Antonella Brunello, Fabio Gelsomino, Tommaso De Pas, Toni Ibrahim, Lorena Gurrieri, Federica Grosso, Francesca Zanelli, Maria Abbondanza Pantaleo, Laura Milesi, Libero Ciuffreda, Vittorio Ferrari, Emanuela Marchesi, Irene Quattrini, Alberto Righi, Elisabetta Setola, Elisa Carretta, Paolo G. Casali, Piero Picci, Stefano Ferrari

**Affiliations:** ^1^ Osteoncology, Bone and Soft Tissue Sarcomas and Innovative Therapies Unit IRCCS Istituto Ortopedico Rizzoli, Bologna, Italy; ^2^ Medical Oncology Unit 2, Fondazione IRCCS Istituto Nazionale dei Tumori, Milano, Italy; ^3^ Candiolo Cancer Institute, FPO-IRCCS, Candiolo (TO), Italy; ^4^ Centro di Riferimento Oncologico di Aviano (CRO Aviano), IRCCS, Aviano, Italy; ^5^ Oncology Unit, Azienda Ospedaliero-Universitaria Pisana, Pisa, Italy; ^6^ Department of Surgical, Oncological and Oral Sciences, Section of Medical Oncology, University of Palermo, Palermo, Italy; ^7^ Sarcomas and Rare Tumors Unit, Sarcomas and Rare Tumors Departmental Unit-IRCCS Regina Elena National Cancer Institute, Rome, Italy; ^8^ Medical Oncology, University Campus Bio-Medico, Rome, Italy; ^9^ Struttura Complessa (SC) Oncologia ASL Città di Torino, Ospedale San Giovanni Bosco, Torino, Italy; ^10^ Sarcomas and Rare Tumors Unit, Istituto Nazionale Tumori IRCCS “Fondazione G. Pascale”, Naples, Italy; ^11^ Department of Oncology, Medical Oncology 1, Veneto Institute of Oncology IOV-IRCCS, Padua, Italy; ^12^ Department of Oncology and Hematology, University Hospital of Modena, Modena, Italy; ^13^ Unit of Medical Oncology Sarcomas, Thymomas and Rare Tumors, European Institute of Oncology, IRCCS, Milano, Italy; ^14^ Osteoncology and Rare Tumors Center, IRCCS Istituto Romagnolo per lo Studio dei Tumori (IRST) “Dino Amadori”, Meldola, Italy; ^15^ Mesothelioma and Rare Cancer Unit, Azienda Ospedaliera SS. Antonio e Biagio General Hospital, Alessandria, Italy; ^16^ Dipartimento Oncologico e Tecnologie Avanzate, Arcispedale Santa Maria Nuova IRCCS Reggio Emilia, Reggio Emilia, Italy; ^17^ Division of Oncology, IRCSS Azienda Ospedaliero-Universitaria di Bologna, Bologna, Italy; ^18^ Department of Oncology, ASST. Papa Giovanni XXIII Hospital, Bergamo, Italy; ^19^ Medical Oncology Unit, Azienda Ospedaliero Universitaria San Giovanni Battista, Molinette, Torino, Italy; ^20^ Dipartimento di Specialità Medico-Chirurgiche, Scienze Radiologiche e Sanit Sanità Pubblica, Oncologia Medica, Università degli Studi di Brescia, ASST Spedali Civili, Brescia, Italy; ^21^ Italian Sarcoma Group Clinical Trial Unit, IRCCS Istituto Ortopedico Rizzoli, Bologna, Italy; ^22^ Department of Pathology, IRCCS Istituto Ortopedico Rizzoli, Bologna, Italy; ^23^ Department of Experimental, Diagnostic and Speciality Medicine, Alma Mater Studiorum, University of Bologna, Bologna, Italy; ^24^ Department of Oncology and Hemato-oncology, University of Milan, Milan, Italy

**Keywords:** sarcoma, soft tissue, rare, ultra-rare, translocation-related

## Abstract

**Background:**

Among sarcomas, which are rare cancers with an incidence of <6 per 100.000/year cases, ultra-rare sarcomas have an incidence of approximately ≤1/1,000,000/year cases and altogether account for ~20% of all soft tissue sarcomas (STS) and bone sarcomas. The Italian Sarcoma Group has recently performed a non-interventional, retrospective TrObs study with data from 512 anthracycline-pretreated patients with advanced multiple STS histologies and treated with trabectedin (Palmerini, *Cancers* 2021; ClinicalTrials.gov Identifier: NCT02793050).

**Methods:**

A *post-hoc* analysis of case series to evaluate the efficacy and safety of trabectedin on patients with ultra-rare and other rare translocation-related sarcomas included in TrObs study was performed. Main outcomes comprised investigator-assessed overall response rate (ORR), disease control rate (DCR), progression-free survival (PFS) and safety.

**Results:**

Thirty-six patients (18 women) with ultra-rare and other rare sarcoma and a median age of 53.0 years (range: 22-81) were included. Most patients had solitary fibrous tumor (SFT; n=11) followed by epithelioid sarcoma (n=5), malignant peripheral nerve sheath tumor (MPNST; n=4), extraskeletal myxoid chondrosarcoma (EMC; n=3), desmoplastic small round cell tumor (DSRCT; n=3), and alveolar soft part sarcoma (ASPS), rhabdomyosarcoma and clear cell sarcoma (n=2 each). Thirty-five patients had metastatic disease and 23 patients received trabectedin as a second-line treatment. Among 35 patients evaluable for response, two patients with SFT and ASPS had a partial response and one patient with DSRCT obtained a complete response, reaching an ORR of 8.6% (95% CI: 2.8-23.4%). Among patients with an ORR, 6-months PFS was 100% in patients with ASPS, 45.7% in patients with SFT and 33.3% in those with DSRCT. Two patients with epithelioid sarcoma and myoepithelioma had disease stabilization lasting >24 months. Nine patients had at least one grade 3/4 adverse event, mostly being bone marrow toxicity (n=6).

**Conclusions:**

Trabectedin has some anti-tumor activity in some ultra-rare and other rare sarcomas, particularly translocation-related sarcomas, with the well-known manageable safety profile.

## Introduction

Currently the existence of ~100 different sarcomas and mesenchymal tumors of intermediate malignancy is well acknowledged, and each of these entities is marked by a specific morphology, biology, natural history, sensitivity to medical agents, and prognosis (1–3). Among sarcomas, which are rare cancers with an incidence of <6 per 100.000/year cases, many types are exceedingly rare. As recently agreed under the umbrella of the Connective Tissue Oncology Society (CTOS), ultra-rare sarcomas are considered those with an annual incidence of approximately ≤1 per 1.000.000 cases (4). Based on this threshold, a list of ultra-rare sarcomas was defined, including 56 soft tissue sarcoma (STS) types and 21 bone sarcoma types, which roughly accounts for 20% of all sarcomas.

Trabectedin (Yondelis^®^, PharmaMar, Spain) is a semi-synthetic drug originally isolated from the sea squirt *Ecteinascidia turbinata*. Trabectedin binds to the minor groove of DNA and blocks DNA repair machinery, and its pleiotropic mechanisms of action include induced direct growth inhibition and death of malignant cells, modulation of inflammatory responses in the tumor microenvironment and inhibition of the factors that promote tumor growth, angiogenesis, and metastasis (5–7). Moreover, trabectedin is considered particularly effective against translocation-related sarcomas, since it modulates the transcription of the oncogenic fusion proteins (8–12). Trabectedin was the first anticancer marine-derived drug approved in the European Union in 2007 and currently in nearly 80 countries around the globe for the treatment of adults with advanced STS after failure of anthracycline and ifosfamide, or for those patients who are unsuited to receive these agents (13). Since 2015, following the analysis of a pivotal, active-controlled, randomized phase III trial in patients with advanced liposarcoma or leiomyosarcoma (commonly abbreviated as L-sarcomas) after failure of prior anthracycline-containing chemotherapy, trabectedin was also approved by the U.S. Food and Drug Administration (14, 15). Trabectedin is also active in some non-L-sarcomas (16–18).

The Italian Sarcoma Group has recently carried out a non-interventional, retrospective TrObs study (ClinicalTrials.gov Identifier: NCT02793050) with data from pretreated patients with advanced sarcoma of multiple histologies and treated with trabectedin (19). That study provided additional insights of the real-world efficacy, toxicity and management of patients treated with trabectedin in clinical practice across Italy. Herein, building on the data from patients with ultra-rare and other rare STS included in the TrObs study, we carried out a *post-hoc* case series analysis to evaluate the efficacy and safety of trabectedin only in such a patient population.

## Methods

### TrObs study

Full details of TrObs study have been reported earlier (19). Briefly, TrObs (**Tr**abectedin in Soft Tissue Sarcomas: A Retrospective **Obs**ervational Analysis) was a non-interventional, retrospective study that evaluated trabectedin in routine clinical practice across Italy. Patients with advanced STS were treated with trabectedin in accordance with the marketing authorization and local clinical practice. The primary endpoint was to describe the clinical characteristics of patients treated with trabectedin, whereas main secondary endpoints included objective response rate (ORR) according to treating physician evaluation based on the Response Evaluation Criteria in Solid Tumors (RECIST) version 1.1 (20), the disease control rate (DCR) and the assessment of progression-free survival (PFS), overall survival (OS) and safety. The ORR was defined as the percentage of patients who achieved a complete (CR) or partial response (PR), whereas DCR was defined as the percentage of patients with a radiological CR, PR or stable disease (SD). The study was conducted in 512 patients enrolled in 20 Italian recruiting sites from January 2010 to December 2015.

### *Post-hoc* analysis

A *post-hoc* analysis was carried out to evaluate the ORR, DCR, PFS and safety in case series with ultra-rare and other rare translocation-related sarcoma (excluding L-sarcomas, synovial sarcoma and undifferentiated pleomorphic sarcoma) following the treatment with trabectedin in TrObs study. Univariate analyses of PFS was performed using the following prognostic factors: sarcoma histology, age, number of prior lines of chemotherapy, response to trabectedin, prior radiotherapy and existence of surgically free disease.

All study procedures were carried out in accordance with the Declaration of Helsinki and its later amendments and local regulations on clinical trials, and were approved by the institutional review boards of each participating center. Due to the de-identified nature of the data collected in this study, signed informed consents were obtained from all alive study participants at enrolment.

## Results

This *post-hoc* analysis included 36 patients (18 women) with ultra-rare (n=20) or other rare sarcoma (**Table 1**). Patients had a median age of 53.0 years (range: 22-81) and an ECOG performance status score of 0/1 was recorded in 30 patients (83.3%). Among patients with rare sarcoma, most patients had solitary fibrous tumor (SFT; n=11) following by malignant peripheral nerve sheath tumor (MPNST; n=4), and dermatofibrosarcoma protuberans with fibrosarcomatous changes (DFSP-FS) (DFSP; n=1). Among those with an ultra-rare sarcoma, epithelioid sarcoma (n=5), extraskeletal myxoid chondrosarcoma and desmoplastic small round cell tumor (EMC and DSRCT; n=3) were the most frequent diagnosis, alveolar soft part sarcoma (ASPS), rhabdomyosarcoma and clear cell sarcoma were found in two patients each, whereas malignant myoepithelioma, epithelioid hemangioendothelioma, epithelioid leiomyosarcoma were remaining diagnosis in one patient each. The most common site of the primary tumor was lower extremity (n=10) and intra-abdominal (n=4). Nearly all patients had metastatic disease (n=35), mostly being lung metastases (n=22), 23 patients (63.9%) had surgically free disease. Four patients had only lung metastases, while two and ten patients had single-organ bone and other metastases. Overall, 11 patients received prior radiotherapy and most patients were pretreated with one chemotherapy line (n=23; 63.9%) prior to trabectedin administration (**Table 1**).

**Table 1:** Patient and disease characteristics at baseline.

Patients (n)	Enrolled patients; n=36	
**Gender**	Male	18 (50.0%)
	Female	18 (50.0%)
**Age at study entry (years)**	Median	53.0
	Range (Min-Max)	22.0-81.0
**Age group**	≤60	27 (75.0%)
	>60	9 (25.0%)
**Ultra-rare sarcomas**	Epithelioid sarcoma	5 (13.9%)
	Extraskeletal myxoid chondrosarcoma (EMC)	3 (8.3%)
	Desmoplastic small round cell tumor (DSRCT)	3 (8.3%)
	Rhabdomyosarcoma	2 (5.6%)
	Alveolar soft part sarcoma	2 (5.6%)
	Clear cell sarcoma	2 (5.6%)
	Malignant myoepithelioma	1 (2.8%)
	Epithelioid hemangioendothelioma	1 (2.8%)
	Epithelioid leiomyosarcoma	1 (2.8%)
**Rare sarcomas**	Solitary fibrous tumor (SFT)	11 (30.6%)
	Malignant peripheral nerve sheath tumor (MPNST)	4 (11.1%)
	Dermatofibrosarcoma protuberans with fibrosarcomatous changes (DFSP-FS)	1 (2.8%)
**Site of primary tumor at first diagnosis**	Lower extremity	10 (27.8%)
	Intra-abdominal	4 (11.1%)
	Trunk	3 (8.3%)
	Visceral	3 (8.3%)
	Thoracic (non-lung)	3 (8.3%)
	Head and neck	3 (8.3%)
	Thoracic (lung)	2 (5.6%)
	Upper extremity	2 (5.6%)
	Visceral gynecological	2 (5.6%)
	Sacrum level	1 (2.8%)
	Missing	3 (8.3%)
**Eastern Cooperative Oncology Group (ECOG) performance status**	0	18 (50.0%)
	1	12 (33.3%)
	2	4 (11.1%)
	Missing	2 (5.6%)
**Tumor stage at study entry**	Locally advanced	1 (2.8%)
	Metastatic	35 (97.2%)
	Lung metastases	22 (61.1%)
	Bone metastases	10 (27.8%)
	Other metastases	26 (72.2%)
**No. of lines of prior chemotherapy**	1 line	23 (63.9%)
	2 lines	5 (13.9%)
	≥3 lines	8 (22.2%)

Among 36 patients, one patient had missing data; therefore, 35 patients were evaluable for response. Two patients with SFT and ASPS had a PR and one patient with DSRCT obtained a CR, reaching the ORR of 8.6% (95% CI: 2.8-23.4%). Twelve patients had SD as a best response for a DCR of 42.9%. The ORR and DCR per each sarcoma histology are detailed in **Table 2**.

**Table 2:** Response assessment of trabectedin.

All evaluable patients (n=35*)	ORR	Stable disease	DCR	Progressive disease	Total patients
**n (%)** **95% CI**	3 (8.6%) 2.8-23.4%	12 (34.3%)	15 (42.9%) 27.7-59.4%	20 (57.1%)	35 (100.0%)
**Best response by histology (%)**					
SFT	1 (9.1%)	4 (36.4%)	5 (45.5%)	6 (54.5%)	11 (31.4%)
Epithelioid sarcoma	–	3 (60.0%)	3 (60.0%)	2 (40.0%)	5 (14.3%)
MPNST	–	1 (25.0%)	1 (25.0%)	3 (75.0%)	4 (11.4%)
EMC	–	2 (66.7%)	2 (66.7%)	1 (33.3%)	3 (8.6%)
DSRCT	1 (33.3%)	–	1 (33.3%)	2 (66.7%)	3 (8.6%)
Clear cell sarcoma	–	–	–	2 (100.0%)	2 (5.7%)
Rhabdomyosarcoma	–	–	–	2 (100.0%)	2 (5.7%)
Alveolar soft part sarcoma	1 (50.0%)	1 (50.0%)	2 (100.0%)	–	2 (5.7%)
Epithelioid leiomyosarcoma	–	–	–	1 (100.0%)	1 (2.9%)
Hemangioendothelioma	–	–	–	1 (100.0%)	1 (2.9%)
Myoepithelioma	–	1 (100.0%)	1 (100.0%)	–	1 (2.9%)

*One patient has missing data.

DCR; disease control rate; DSRCT, desmoplastic small round cell tumor; EMC, extraskeletal myxoid chondrosarcoma; MPNST, malignant peripheral nerve sheath tumor; ORR; objective response rate; SD; stable disease; SFT, solitary fibrous tumor

**Figure 1:**
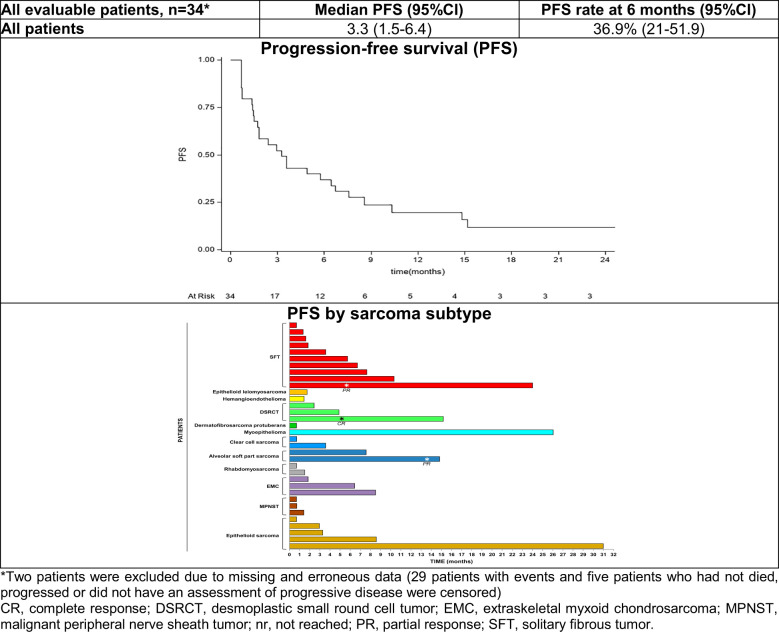
Kaplan-Meier plots of progression-free survival.

After a median follow-up of 24.0 months (interquartile range: 8.5-26.0 months) 34 patients were evaluated for PFS as two patients were excluded due to missing data. Median PFS was 3.3 months (95% CI: 1.5-6.4) with 36.9% of patients free from progression at 6 months (**Figure 1**). Noteworthy, among patients with an ORR, 6-month PFS was 100% in patients with ASPS, 45.7% in patients with SFT and 33.3% in those with DSRCT (**Table 3**). Two patients with epithelioid sarcoma and myoepithelioma had disease stabilization lasting >24 months. Significantly longer median PFS and higher PFS rates at 6-months were observed in patients who obtained objective responses or SD as compared with patents with progressive disease (*p*<0.001). We also observed a clear trend towards longer PFS in patients with surgically free disease as compared to those with non-surgically free (*p*<0.0721) (**Table 3**).

**Table 3:** Univariate analyses of progression-free survival.

Univariate analyses of Progression-free survival (PFS)			
PFS by histology in patients with objective response	Median PFS	PFS at 6 months	Log-rank test
Alveolar soft part sarcoma	11.2 (7.6-nr)	100%	0.9358
Solitary fibrous tumor	5.7 (0.7-nr)	45.7% (14.3-73)	
Desmoplastic small round cell tumor	4.9 (2.4-nr)	33.3% (9.0-77.4)	
**PFS by age**			
≤60y	2.4 (1.4-6.4)	32% (15.2-50.2)	0.2037
>60y	6.7 (0.7-nr)	51.9% (16.4-78.8)	
**PFS by prior chemotherapy line**			
1	4.2 (1.3-8.6)	40.9% (20.9-60.1)	0.6941
>1	3 (0.7-6.4)	28.6% (7-55.5)	
**PFS by best response**			
Objective response rate (ORR)	15.2 (14.8-nr)	100%	0.0002 (ORR *vs.* SD *vs.* PD)
Stable disease (SD)	7.6 (3.0-nr)	63.6% (29.7-84.5)	0.0027 (ORR *vs.* PD)
Progressive disease (PD)	1.7 (0.7-3.6)	11.7% (2.0-30.9)	0.0008 (SD *vs.* PD)
			0.3840 (ORR *vs.* SD)
**PFS by surgically free disease**			
Non-surgically free	2.4 (0.7-4.9)	18.2% (2.9-44.2)	0.0721
Surgically free	5.7 (1.5-10.3)	45.9% (23.9-65.5)	
**PFS by prior radiotherapy**			
No prior radiotherapy	3.5 (1.3-8.6)	39% (18.9-58.6)	0.7922
Prior radiotherapy	3.1 (0.7-6.4)	30% (7.1-57.8)	

Nine patients had at least one grade 3/4 adverse event, mostly being bone marrow toxicity (n=6; 66.7%) and transaminase increases (n=2; 22.2%), in line with the safety profile on trabectedin in the primary analysis of TrObs study (19). During the study, no drug-related deaths or new or unexpected adverse events were observed. Eighteen patients with ultra-rare or other rare sarcoma received a subsequent antineoplastic treatment.

## Discussion

Ultra-rare STS pose inherent challenges not only for diagnosis but also for appropriate treatment, since their rarity makes it extremely difficult to conduct well-powered prospective clinical studies to support new drug discovery and development. The results of a recent retrospective, single-institution study that reviewed records from patients on phase I trials reported that not only patients with ultra-rare sarcomas responded similarly or better than patients with more common sarcoma diagnoses but also clinically benefited more from molecularly matched treatments as compared with unmatched trials, confirming that genomic selection may help identify molecular subsets likely to benefit from targeted therapy (21). The data from this *post-hoc* case series analysis confirm the activity of trabectedin in a real-life setting, with a manageable well-known safety profile characterized by transient and non-cumulative toxicities of bone marrow suppression and hepatotoxicity (22), among patients with ultra-rare and other rare translocation-related sarcomas. Namely, in our series trabectedin was associated with an interesting disease control rate as well as a significant progression-free interval in selected patients with some rare translocation-related sarcomas. Trabectedin was noted early in clinical development to demonstrate very relevant antitumor activity against myxoid- round cell liposarcoma (MRC-L-sarcoma) (23, 24). The high activity against this type of sarcoma seems to be related to trabectedin ability to counteract the biological activity of the chimeric FUS-DDIT3 oncoprotein, a hallmark of this disease (23). *In vitro*, data showed that trabectedin can remove differentiation blockade mediated by the FUS-DDIT3 chimera and induce adipocytic differentiation (25) and that the expression of different variants of the FUS-DDIT3 fusion transcripts correlates with the sensitivity to trabectedin (26). Considering the likely functional similarities of the fusion oncoproteins of MRC-L-sarcomas with those of other TRS, a retrospective analysis of trabectedin activity in different TRS subtypes was performed (11). This analysis in patients with several TRS subtypes showed 10% ORR, 59% tumor control rate, median PFS of 4.1 months, and PFS rates at 6 months in all TRS (40%) that, as in our study (PFS at 6 months: 36.9%), notably exceeded the cutoff of 14% proposed by the Soft Tissue and Bone Sarcoma Group of the European Organisation for Research and Treatment of Cancer (EORTC-STBSG) to consider a therapy as active in pretreated STS (27, 28). Our results further support the outcomes reported by Kawai et al. who following a phase II randomized comparison study observed that trabectedin reduced the risk of progressive disease or death in patients with advanced translocation-related sarcoma in a second-line setting as compared with best supportive care (median PFS: 5.6 months, 95% CI: 4.1-7.5 *vs.* 0.9 months, 95% CI: 0.7-1.0) (8). All this data from clinical trials, retrospective series and cases reports (8–12, 29) evaluating trabectedin in patients with several TRS subtypes supports the hypothesis that trabectedin might act as an inhibitor of interaction of aberrant transcription factors derived from chromosomal translocations with tumor cell DNA in these sarcoma subtypes.

The key limitation of our case series study is its non-interventional and retrospective nature. In addition, the sample size was very small, with only one or two cases treated with trabectedin for each one of seven ultra-rare histotypes. Nevertheless, while we await data from larger prospective studies, our results provide some evidence that trabectedin is a clinically meaningful and safe option for pre-treated patients with selected ultra-rare and rare sarcomas.

In conclusion, this analysis confirmed the activity of trabectedin in selected ultra-rare and other rare STS histotypes. The responses described in some of these histological types recall evidence that trabectedin may be particularly active in some translocation-related sarcoma and warrant further histotype-specific prospective studies.

## Data availability statement

The original contributions presented in the study are included in the article/supplementary material. Further inquiries can be directed to the corresponding author.

## Ethics statement

All study procedures were carried out in accordance with the Declaration of Helsinki and its later amendments and local regulations on clinical trials and were approved by the institutional review boards of each participating center. Due to the retrospective and de-identified nature of the data collected in this study, signed informed consents were obtained from all alive study participants at enrolment. The patients/participants provided their written informed consent to participate in this study.

## Author contributions

Conception/design: EP, RS, GG, ABu, PC, PP, and SF. Provision of study material/patients: EP, RS, GG, ABr, and PC. Collection and/or assembly of data: All authors. Data analysis and interpretation: All authors. All authors contributed to the article and approved the submitted version.
